# A stab in the back with a screwdriver: a case report

**DOI:** 10.1186/1757-1626-1-305

**Published:** 2008-11-11

**Authors:** Mohammed A Bhutta, Paul D Dunkow, Derick M Lang

**Affiliations:** 1 North Western Deanery Trauma and Orthopaedics, South Manchester University Healthcare Trust, UK; 2 Department of Trauma and Orthopaedics, Blackpool Flyde and Wyre NHS Trust, UK; 3 Department of Trauma and Orthopaedics, South Manchester University Healthcare Trust, UK

## Abstract

**Background:**

Stabbings infrequently produce spinal injury. However, the use weapons other than blades can overcome this natural defence barrier.

**Case Presentation:**

We present a spinal injury produced by a screwdriver, its management and a review of the literature.

**Conclusion:**

This case highlights the need for clinical vigilance, including in those who appear stable and a senior multidisciplinary approach to each individual case.

## Background

Injuries of the spine as a result of direct stabbings are infrequent outside of South Africa from where previous literature has been produced. During such assaults the injury is often inflicted from behind, where normally blades are deflected away from the spinal cord. However, there is increasing use of alternative weapons to hide any criminal intent such as screwdrivers. These implements are less likely to break or be deflected and are capable of penetrating bone.

## Case presentation

A 36 year old male was found lying on his back on a road by police in the early hours of the morning. He was confused and unable to recollect events but did not appear intoxicated. Minor soft tissue injuries were visible, but as a road traffic incident could not be excluded a paramedic unit placed him on a spinal board at the scene.

In the Accident and Emergency department, ATLS guidelines were followed where the airway, breathing and circulation where normal. The patient was now coherent with a GCS 15/15 and neurologically intact.

**Figure 1:**
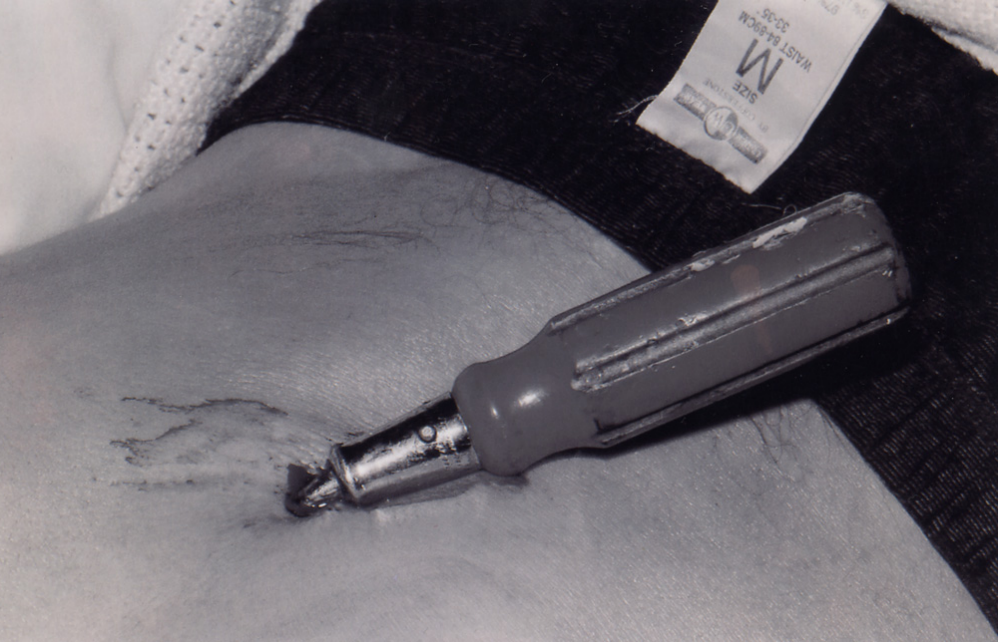
Photograph of stabbing while in the emergency room.

The patient continued to complain of facial, skull and back pain. Facial lacerations, abrasions and bruising could be seen. However, when he was log-rolled the cause for his back pain was found. A screwdriver 'inserted' to the hilt at which there was a 90 degree bend (Figure 1).

**Figure 2:**
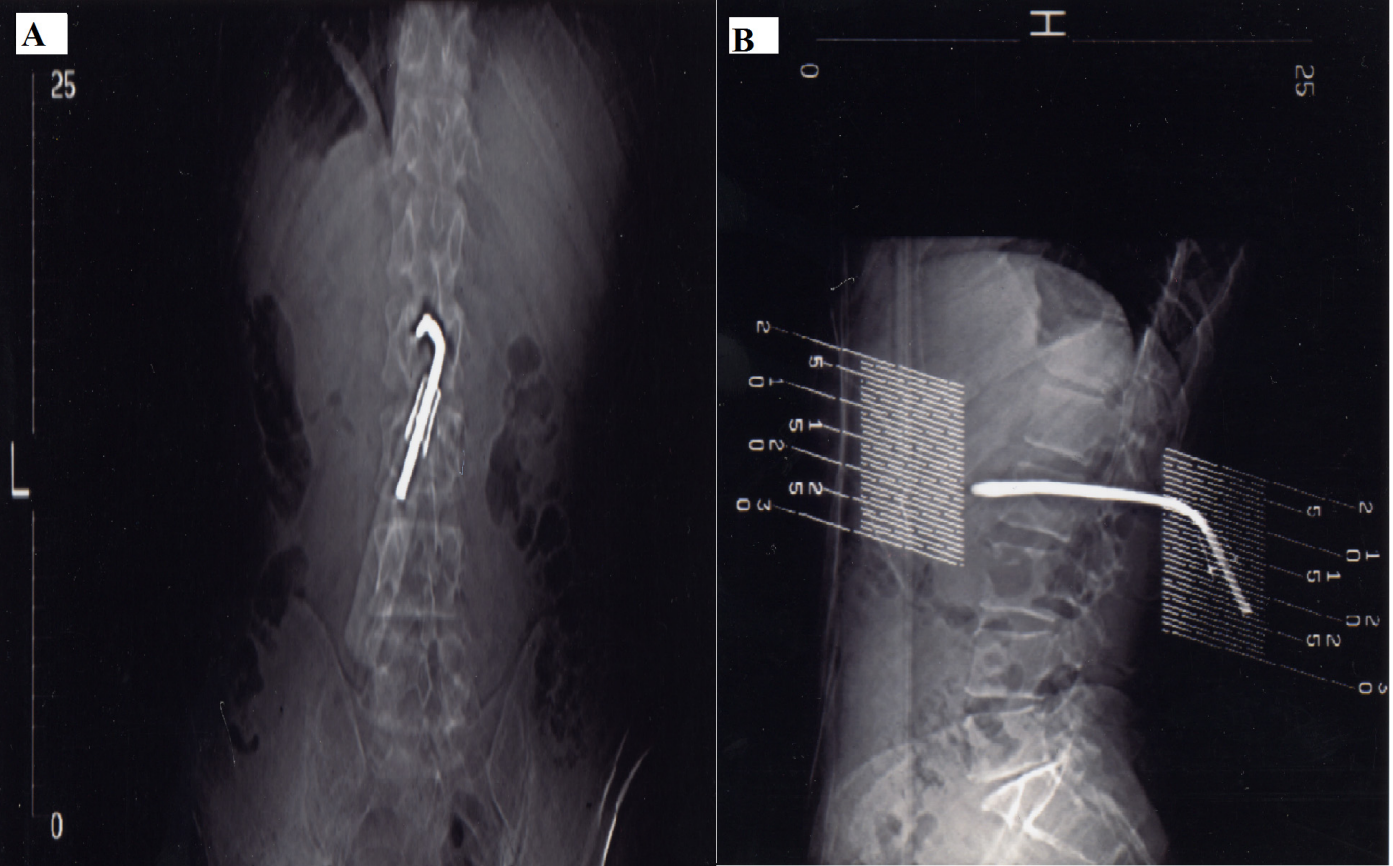
(A) AP and (B) Lateral CT scan of abdomen demonstrating position of screwdriver.

**Figure 3:**
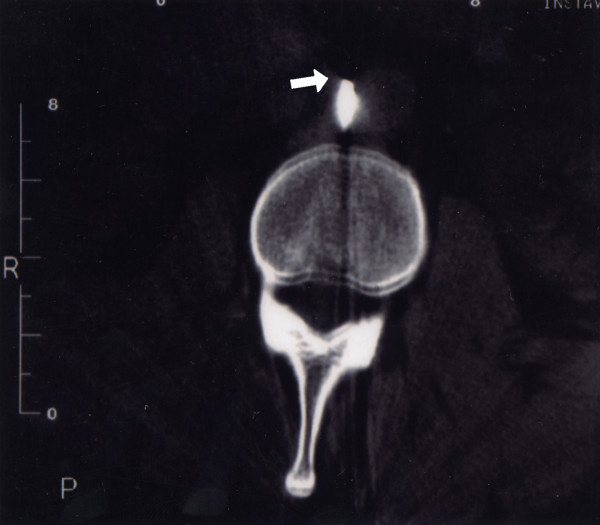
CT scan demonstrating tip of screwdriver abutting the abdominal aorta.

A secondary survey did not reveal any other significant injuries. Initial plain X-rays taken demonstrated a 12 cm screwdriver at the 2^nd ^lumbar vertebrae protruding beyond the vertebral body by approximately 2 cm. After discussion with a radiologist, a CT scan was performed and confirmed the screwdriver entering at the level of L2, right of the spinous process penetrating the lamina, traversing the spinal canal and body of L2. It's path missing the filum terminale and the tip abutting the abdominal aorta. No evidence of haemorrhage was seen (Figure 2, 3).

A multidisciplinary team of orthopaedic and vascular surgeons, anaesthetist and the patient were involved in the management decision. This was to remove the screwdriver under local anaesthetic with mild sedation in an operating theatre, thus allowing observations for any change in neurology, a vascular team was also scrubbed and ready on standby.

The screwdriver was successfully removed. The patient was given prophylactic antibiotics and discharged 3 days later. A follow-up Magnetic Resonance Imaging scan three months later revealed mild arachidonitis, with no thecal disruption and the patient remained clinically well.

## Discussion

Stabbings involving the back are relatively common, accounting for 12% of all stabbing fatalities in the UK, with a much higher incidence in other countries [1,2]. Literature shows a higher incidence of stabbing in young men, with most to the thoracic spine (61%) and least to the lumbar spine (7%) [1].

Screwdrivers, however, are infrequently used in such assaults, and the few incidences reported are of varying intracranial injuries producing unique signs and symptoms [3-6]. As a weapon it is able to apply a concentrated force to a small area at the tip with a strong stem allowing it to penetrate bone as compared to a blade which tends to snap or slide. As for most stabbings wounds may be small externally but can often mask an arc of damage internally, which may not manifest itself immediately. Therefore, there is a need for a high index of suspicion even in patients who are haemodynamically stable.

Direct central back stabbings however, rarely produce injuries to the spinal cord and central retroperitoneal structures due to the protection provided by the layers of muscle and the spinal column, with the spinous and transverse processes deflecting blades laterally [7,8]. In this case the unusual weapon and mechanism of injury were able to permeate this defence. On review of the imaging (Figures 2, 3) we postulate that during the assault, the assailant most likely thrust the weapon into the L2 lamina with partial penetration of bone. Following this the victim either collapsed or was pushed backwards resulting in the screwdriver being driven deeper, potentially exposing the spinal cord and retroperitoneal structures. The patient was extremely fortunate to avoid neural or vascular damage.

There is much debate regarding the management of stab wounds to the back/spine and flank. In a clinically stable patient, there is a trend towards non-operative management, with the use of triple contrast CT scan as a tool to ensure no serious sequale have been missed. Although this does not affect the cost per patient, they spend less time in hospital [7,8]. The CT scan can also elucidate the track of the injury and aid any surgery that maybe required. In our case a plain CT was performed given the direct spinal penetration of the screwdriver visualised from plain radiographs, decreasing the probability of intestinal injury. The CT scan revealed the path of the screwdriver and that the tip was abutting the abdominal aorta. No focal haemorrhage was seen, and in this instance an arc of injury was not produced since the screwdriver was embedded in the lumbar vertebrae. Indications for laparotomy or exploration in a stable patient include a retained foreign body, radiological compression of the spinal cord, spinal cord herniation [9]. Other complications which can occur are cerebrospinal fluid leak (occurring in up to 6% of injuries [6]), local abscess formation, osteomyelitis, pnemomyelogram and damage to the artery of Adamkiewicz [10].

## Conclusion

This case report demonstrates an unusual injury produced by a screwdriver to the spine without neurological, vascular or bony consequence. It highlights the need for clinical vigilance in all trauma patients, even in those who appear stable. We would in retrospect recommend the use of CT scans with contrast to delineate occult injuries. It also demonstrates the use of a common sense approach within a senior multidisciplinary team to formulate the best management plan in each unique case.

## Consent

"Written informed consent was obtained from the patient for publication of this case report and accompanying images. A copy of the written consent is available for review by the Editor-in-Chief of this journal."

## Competing interests

The authors declare that they have no competing interests.

## Authors' contributions

MAB major contributor to writing of manuscript. PDD contributor to writing and editing manuscript. DL senior author and manager of case and final editor.
